# Psychological Effects of Aromatherapy on Smokers With Depressive Tendencies During Smoking Cessation Treatment: Protocol for a Pre-Post Single-Arm Clinical Trial

**DOI:** 10.2196/38626

**Published:** 2022-07-07

**Authors:** Akiko Hata, Maki Komiyama, Akihiro Yasoda, Hiromichi Wada, Hajime Yamakage, Noriko Satoh-Asahara, Tatsuya Morimoto, Yuko Takahashi, Koji Hasegawa

**Affiliations:** 1 Clinical Research Institute National Hospital Organization Kyoto Medical Center Kyoto Japan; 2 Division of Molecular Medicine School of Pharmaceutical Sciences University of Shizuoka Shizuoka Japan; 3 Health Informatics Faculty of Medicine Kyoto University Kyoto Japan

**Keywords:** smoking cessation, aromatherapy, depression, cardiovascular risk, inhaler, complementary and alternative medicine

## Abstract

**Background:**

Cessation of smoking can markedly reduce the incidence of cardiovascular disease, improve health economics, and benefit society. Aromatherapy has the potential to be a novel option as an adjuvant therapy for smoking cessation that may alleviate depressive symptoms. However, research on the efficacy of aromatherapy as an adjuvant therapy for smoking cessation is scarce.

**Objective:**

The aim of this study was to examine the potential effects of aromatherapy on psychological states in smokers with depressive tendencies and to determine if it is reasonable to proceed to the next step (ie, a phase III trial).

**Methods:**

This is a pre-post single-arm clinical trial. Smokers with depression will be subjected to aromatherapy during smoking cessation treatment for 12 weeks. We will evaluate changes in scores on the Zung Self-Rating Depression Scale and the Profile of Mood States from pretreatment screening to 4 weeks and 12 weeks after the start of aromatherapy. Moreover, we will compare the group treated with aromatherapy with the group that received standard treatment in our previous randomized controlled trial (ie, the control group in that study). Furthermore, we will compare successful smoking cessation rates after 12 weeks. In addition, we will conduct an exploratory analysis of the efficacy of aromatherapy. The target sample size is 100, which is the number of subjects expected to be enrolled in this study during the 2-year study period.

**Results:**

This study was approved by the Kyoto Medical Center Institutional Review Board (IRB approval No. 19-016). Enrollment started on July 1, 2019. As of May 2022, 76 patients have been recruited. In the original plan, recruitment should have been finished on June 30, 2021. However, the number of subjects decreased due to the COVID-19 pandemic, and the study inclusion period was extended by 1 year (ie, until the end of June 2022) with the approval of the IRB on May 17, 2021. Analyses of the results will be completed subsequently.

**Conclusions:**

This study has some limitations. This is not a rigorous validation study because it compares the same subjects who received standard treatment in a previous study. Moreover, the sample size and methods of statistical analysis were not fully set with prior consideration of statistical rigor. To address these limitations, we plan to conduct a phase III trial that will reflect the exploratory findings of this study. This is the first study to evaluate the psychological effects of aromatherapy during a smoking cessation program, and it may help improve the quality of treatment for smoking cessation in the future.

**Trial Registration:**

UMIN Clinical Trials Registry UMIN000043102; https://tinyurl.com/tn3hvt9w

**International Registered Report Identifier (IRRID):**

DERR1-10.2196/38626

## Introduction

### Psychological Effects of Smoking

Smoking is a major risk factor for noncommunicable diseases (NCDs), such as cancer, chronic obstructive pulmonary disease, diabetes, cerebral infarction, and myocardial infarction [1]. There is an urgent need for active guidance on smoking cessation, not only to reduce the risk of developing NCDs, but also from social and health economics perspectives. Nicotine patches and varenicline are widely used as pharmacotherapies for smoking cessation, as they are known to increase the rate of successful smoking cessation by 2 to 5 times [2-4].

Smoking is known to cause or exacerbate negative emotions [5]. Moreover, the risk of developing depression is higher among smokers than among nonsmokers [6,7]. In addition, smokers with mental disorders show changes in behavior, agitation, and depressed mood after starting smoking cessation treatment, and the rate of outpatient psychiatric visits increases due to worsening of these symptoms [8]. In patients with no mental disorders at the start of smoking cessation treatment, major depression was reported to have occurred in 14.1% of the patients within 12 months of starting treatment [9]. It is well known that nicotine withdrawal can cause depressive symptoms [10], and the Standard Procedure Manual for Smoking Cessation Treatment (version 8.1) states that smoking cessation, with or without treatment, may be associated with a variety of symptoms and may exacerbate underlying mental disorders [11]. Hence, it can be argued that it is essential to adopt a comprehensive approach considering the close relationship between smoking and psychosocial stress, because smoking cessation can lead to temporal worsening of a depressive state and failure to quit smoking, even in those without a history of mental disorders.

### Aromatherapy

Aromatherapy involves the use of essential oils extracted from flowers, leaves, seeds, pericarps, and resins for the treatment and prevention of diseases, physical and mental health, relaxation, and stress relief [12]. Aromatherapy includes methods such as the inhalation of aromas and massage using essential oils diluted in vegetable oils [13]. Among aromatherapy methods, inhalation of essential oils has the advantage of being simple and noninvasive [14]. Once the olfactory cells receive the aroma components of essential oils, sensory information is transmitted from the olfactory nerve to the hypothalamus, which, in turn, acts on the autonomic nervous system via the brainstem. Furthermore, by influencing emotions via the limbic system [15-17], aromatherapy has been shown to alleviate anxiety and depression [18]. This has also been shown to improve sleep quality [19], alleviate fatigue [20,21] and perceived stress [22], and improve cognitive function [23].

### Existing Research on Aromatherapy for Smoking Cessation

Regarding the efficacy of aromatherapy as an adjuvant therapy for smoking cessation, it has been reported that black pepper reduces the urge to smoke [24], and peppermint has been reported to help alleviate respiratory symptoms, such as sputum and cough associated with smoking cessation [25]. Moreover, it has also been reported that various olfactory stimuli, including essential oils, can reduce the urge to smoke [26]. However, high-quality research on this topic is scarce. Furthermore, although it was not a study on treatment for smoking cessation, a systematic review of the efficacy of aromatherapy on depression concluded that it reduces the propensity for depression [18]. In a study in which aromatherapy was applied as an adjuvant therapy, lavender and rosemary were reported to have an anxiolytic effect after smoking cessation [25]. Another study suggested that lavender has anxiolytic and antidepressant-like effects, which are thought to be mediated by the modulation of the N-methyl-D-aspartate receptor and serotonin transporters [27]. Furthermore, exposure to limonene by inhalation, which is found in citrus essential oil, has been reported to increase dopamine levels in the brain [28], and a mechanism of action has been reported for peppermint, suggesting therapeutic efficacy against depression [29]. Furthermore, aromatherapy has been shown to relieve depression, and inhalation is presented as a particularly effective method [30].

### Use of Inhalers as an Adjuvant Therapy for Smoking Cessation

There are various aromatherapy devices, such as aromatherapy inhalers, which are simple and cost-effective for incorporation into daily life. An aromatherapy inhaler is a portable, plastic, lipstick-like container that houses a cotton wick soaked in essential oils. The inhaler enables users to smell the aromas without getting their hands dirty or spilling the oils. Similar “aromasticks” have been used in British hospitals to control symptoms of nausea, insomnia, and anxiety, and to induce relaxation [31,32]. We assume that if aromatherapy inhalers can alleviate psychological and depressive symptoms and reduce cravings for cigarettes, they can form the basis of a novel adjuvant therapy for smoking cessation.

### Hypothesis and Objective

Depression may temporarily worsen during smoking cessation treatment [11]. This study hypothesizes that aromatherapy may help improve this depressive state during treatment. This study aims to examine the effect of adding aromatherapy to standard smoking cessation treatment in patients with depressive tendencies who visited a smoking cessation clinic and to compare these to a historical control group (ie, standard treatment group).

## Methods

### Study Design

This is a pre-post single-arm clinical trial conducted at the National Hospital Organization Kyoto Medical Center in Japan. Patients in this study who visited the smoking cessation clinic at the medical center to receive smoking cessation treatment were assessed using the Zung Self-Rating Depression Scale (SDS) and were classified into two groups: the healthy group (SDS score ≤38) and the depressed group (SDS score = 39-59). Patients in the depressed group are considered to have depressive tendencies. Patients who provide consent will be included in this study, with a target sample size of 100. Smokers with depression will be subjected to aromatherapy during smoking cessation treatment for 12 weeks. Changes in the SDS score and the Profile of Mood States (POMS) score from pretreatment screening to 4 and 12 weeks after the start of aromatherapy will be evaluated. This study will also compare the aromatherapy group with the standard treatment group from a previous study entitled “A Randomized, Multi-center, Double-Blind, Placebo-Controlled Trial for the Effects of Yokukansan on Depressive or Neurotic Smoking Patients during Smoking Cessation Therapy” (University Hospital Medical Information Network [UMIN] ID: 000027036); this group will act as a control group (ie, historical control group). In both studies, only smokers with SDS scores of 39 to 59 were included in the study, and the study subjects were randomly assigned to either the yokukansan group (ie, yokukansan plus standard treatment) or the control group (ie, standard treatment), with 220 subjects in each group. Changes in SDS and POMS scores from pretreatment screening to 4 and 12 weeks after the start of treatment, as well as the rate of successful smoking cessation, will be evaluated. Furthermore, an exploratory analysis on the efficacy of aromatherapy will also be conducted (eg, analyzing the correlation between essential oil preference and smoker information items at the first visit and the changes in essential oil preferences over time).

### Recruitment

#### Inclusion Criteria

In Japan, smoking cessation treatment is covered by health insurance if it is the patient’s first time undergoing the treatment or if more than 1 year has passed since the last treatment. Accordingly, this study included patients covered by health insurance [11] and who met all of the following criteria based on the diagnosis and tests before starting treatment for smoking cessation:

1. Smokers with nicotine dependence (Fagerström Test for Nicotine Dependence [FTND] score of 5 points or more) who wish to quit smoking.
2. Patients with depression with SDS scores of 39 to 59, based on the self-report questionnaire for assessing propensity to depression.
3. Patients aged 20 to 79 years at the time of the informed consent procedure.
4. Outpatients.
5. Patients whose informed consent has been obtained in writing.

This study aims to include 100 participants in the analysis.

#### Exclusion Criteria

Patients will be excluded from this study if it is determined that they meet any one of the following exclusion criteria based on their diagnosis or test results prior to the start of the study:

1. Patients whose condition is inappropriate for aromatherapy, that is, those suffering from shock, severe disease states, poor respiratory status, feeling of nausea, or vomiting, etc.
2. Patients who habitually practice aromatherapy.
3. Patients who are unable to smell the aromas of essential oils due to olfactory dysfunction.
4. Patients with a history of epileptic seizures.
5. Patients who are currently on medication for a psychiatric or psychosomatic condition.
6. Patients with an SDS score of 53 points or higher, which is indicative of underlying depression and need for psychiatric consult, and those prescribed medication for a psychiatric and psychosomatic condition [33].
7. Patients with current symptoms of drug allergy.
8. Patients who are pregnant, are lactating, or intend to become pregnant during the study period.
9. Other patients who are deemed to be unsuitable according to the attending physician (patients with severe dementia, poor compliance, etc).

#### Withdrawal Criteria

A subject will discontinue their participation in the study if any of the following occurs:

1. If a subject informs us of their withdrawal from the study or requests withdrawal of consent.
2. If the attending physician determines that it is inadvisable for the subject to continue participation in the study due to an adverse event (ie, Grade 3 or higher adverse event according to the Common Terminology Criteria for Adverse Events [CTCAE; version 4.0]) [34].
3. If the attending physician determines that continuation of participation in the study is clinically inappropriate due to the exacerbation of a comorbidity or complication.
4. Death.
5. If the attending physician determines that the subject is ineligible to continue the study.
6. If the subject is unable to visit the medical center where the study is being conducted due to relocation or other reasons.

#### Enrollment and Interventions

The attending physician will provide a complete written explanation and obtain written consent from patients who meet the inclusion criteria. Subsequently, the attending physician will perform subject registration after confirming that the patients meet all the inclusion criteria and none of the exclusion criteria by performing prespecified medical tests.

### Procedures

#### Time Point: Enrollment (4 Weeks Before the Study to the Start of the Study)

An eligibility screen will be conducted and informed consent will be provided to those who are eligible. The following assessments will be carried out: the SDS (ie, the tool that is used for tracking changes in depressive symptoms over time during the course of a study and after treatment [35]) and physical examination findings (ie, height, weight, abdominal circumference, blood pressure, and heart rate). In addition, smoking status and exhaled carbon monoxide (CO) levels will be determined, and subject background data will be collected, including age; sex; medical history; comorbidities; presence or absence of allergies; subjective symptoms; drinking patterns, regarding subjects who drink daily or 3 to 4 times a week, and alcohol consumption, which is monitored and reported in converted values; sleep duration; smoking index (ie, the number of cigarettes smoked per day × years of tobacco use); nicotine dependence, measured by the Tobacco Dependence Screener and the FTND; and the subject’s first medication for smoking cessation.

#### Time Point: Allocation (Day of Treatment Start)

##### Procedures

We will commence the following standard smoking cessation and aromatherapy programs for patients who have provided informed consent. In principle, a sufficient amount of time will be allowed for briefing the subjects to facilitate a complete understanding of the content of this study. Subsequently, their informed consent will be obtained. The maximum duration of obtaining informed consent after briefing is 4 weeks. However, if it is confirmed that the subject has sufficiently understood the content of the briefing, their informed consent may be obtained on the day of the briefing. The standard smoking cessation program was administered according to the Standard Procedure Manual for Anti-Smoking Treatment, which was originally issued in March 2006 by the Japanese Circulation Society, the Japan Lung Cancer Society, and the Japanese Cancer Association. Treatment consists of pharmacotherapy with transdermal nicotine patches and oral varenicline as well as nonpharmaceutical therapy with counseling by doctors and nurses. The patients were examined on their first visit and after 2, 4, 8, and 12 weeks (ie, 3 months) while being treated with transdermal nicotine patches or oral varenicline.

##### Aromatherapy

The following aromatherapy program will be provided in this clinical trial. Subjects will choose from four essential oils: black pepper, lavender, peppermint, and citrus (ie, lemon and grapefruit). These essential oils have been used in previous smoking cessation studies, were reported to have antidepressant and anxiety-reducing effects, and are readily available. Black pepper has been used in previous smoking cessation studies [24], while lavender has antianxiety and antidepressant effects [27]. Moreover, peppermint also has a mechanism of action that suggests a therapeutic effect in people with depression [29]. Limonene is reported to increase dopamine levels in the brain [28], similar to how dopamine is released during smoking [36]; all the essential oils selected for this study contain limonene [37-40]. The aromatherapist will provide subjects with an inhaler filled with their chosen oil; subjects will smell these for 10 to 20 seconds 3 times a day (ie, after breakfast, lunch, and dinner) and at other personally preferred times (ie, when their urge to smoke increases or when they feel anxious). The essential oils and inhalers are produced by Mont Saint Michel, Sanritsu Corporation, Osaka, Japan.

##### Profile of Mood States

We will collect the POMS self-assessment sheets completed by the patients. The short version of the POMS is a tool that facilitates understanding of subjects’ current moods, emotions, and changes, with little effort required from the subjects [41]. Self-assessment tests—the SDS and the POMS—will be completed by the subjects. In case of any omissions or obvious errors detected, the medical staff will ask for the information verbally. 

##### Blood Tests

We will analyze subjects’ baseline clinical data by performing blood tests (ie, blood glucose, hemoglobin A_1c_, low-density lipoprotein cholesterol, high-density lipoprotein cholesterol, triglyceride, uric acid, sodium, potassium, creatinine, glutamic-oxaloacetic transaminase, glutamic-pyruvic transaminase, alkaline phosphatase, g-glutamyl transpeptidase, creatine phosphokinase, C-reactive protein, and complete blood count). We will determine whether or not patients are taking antihypertensive drugs, dyslipidemia drugs, oral diabetes drugs, insulin, sleep medication, or other drugs, specifically anticancer drugs, drugs for hormonal treatment, and steroids.

#### Time Point: 2, 4, 8, and 12 Weeks

We will check for any adverse event and list its grade according to the CTCAE (version 4.0). Aromatherapy adherence status will be rated as follows:

- Good: the aromatherapy inhaler is used nearly every day (approximately 80% of the days)
- Somewhat poor: the aromatherapy inhaler is not used on some days (approximately 60% of the days)
- Poor: the aromatherapy inhaler is not used on most days (approximately 30% of the days).

**Figure 1:**
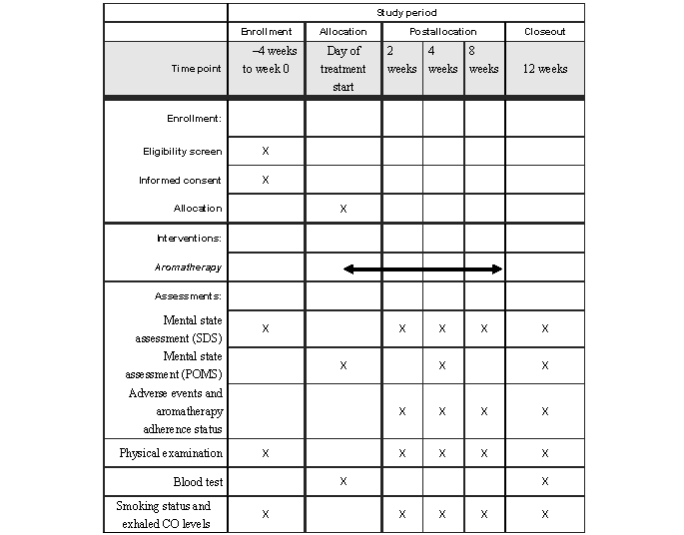
Schedule for observations and tests. "X" indicates that the study task or assessment was performed at the indicated time point. CO: carbon monoxide; POMS: Profile of Mood States; SDS: Zung Self-Rating Depression Scale.

SDS scores, physical examination findings (ie, height, weight, abdominal circumference, blood pressure, and heart rate), smoking status, and exhaled CO levels will also be monitored. Further, in weeks 4 and 12, we will collect the POMS self-assessment sheets completed by the patients. At 12 weeks, we will perform the blood tests and perform a confirmation of smoking cessation. We will determine whether or not patients are taking antihypertensive drugs, dyslipidemia drugs, oral diabetes drugs, insulin, sleep medication, and other drugs, specifically anticancer drugs, drugs for hormonal treatment, and steroids. If a subject does not arrive for a scheduled visit, the reason for their absence will be discussed via a phone call. When the reason is hesitation or the unwillingness of a subject to visit the smoking cessation clinic, they will be requested by medical doctors or nurses to visit the hospital. This strategy is described in the Standard Procedure Manual for Anti-Smoking Treatment, which was originally issued in March 2006 by the Japanese Circulation Society, the Japan Lung Cancer Society, and the Japanese Cancer Association (Figure 1).

#### Study Period

The observation period (ie, follow-up) will last 3 months after the enrollment of the last subject, and the total study period will amount to 2 years and 3 months; the scheduled period was expected to run from July 2019 to September 2021. Since the number of subjects decreased due to the COVID-19 pandemic, the study period was extended to 3 years and 3 months on May 17, 2021, with the approval of the Institutional Review Board (IRB); the study is now expected to run until September 2022.

### Primary Outcome, Secondary Outcomes, and Procedures

#### Primary Outcome

The primary outcome is the change in SDS and POMS scores [35,41] (ie, depression assessment following smoking cessation), which will be evaluated for the period from the pretreatment screening to 4 weeks and 12 weeks after the start of aromatherapy.

#### Secondary Outcomes

The secondary outcomes are as follows:

- Successful smoking cessation rate. The percentage of subjects who stop smoking 12 weeks after aromatherapy (ie, the number of subjects who stop smoking divided by the total number of allocated subjects, as a percentage). Successful smoking cessation refers to a report of smoking cessation for the past 7 days and an exhaled CO level below 7 ppm [42].
- Changes in body weight, abdominal circumference, and glycolipid metabolism (ie, assessment of obesity following smoking cessation and dyslipidemia).
- Adverse events during the period of aromatherapy.
- Blood cell counts and biochemical tests by blood sample collection.
- Other laboratory findings or symptoms.
- Correlation between essential oil preference and smoker information items at first-time visit (ie, day of treatment start). Smoker information items include the following subject background data: age, sex, BMI, number of tobacco pieces consumed or smoked per day, FTND scores, and SDS and POMS scores. These items will be compared among different essential oil types.
- Changes in essential oil preferences over time.

### Statistical Analysis

Research on the efficacy of aromatherapy as an adjuvant therapy for smoking cessation is scarce. Further, based on our research, the size of the effects of aromatherapy on psychological tests as measured by the SDS and the POMS in smokers has never been reported. Therefore, this study was designed as a pre-post single-arm clinical trial in which the sample size has been determined by considering the feasibility of registration. We will perform a comparison of the aromatherapy group with the standard treatment group from our previous study, “A Randomized, Multi-center, Double-Blind, Placebo-Controlled Trial for the Effects of Yokukansan on Depressive or Neurotic Smoking Patients during Smoking Cessation Therapy” (UMIN ID: 000027036). Similar to this aromatherapy study, the yokukansan study targeted the population of “depressed smoking patients.” For the yokukansan study, 100 patients were registered in our hospital.

Assuming that the registration number of this aromatherapy study would be comparable to that of the previous yokukansan study, this study planned to register 100 patients. The SDS scores and POMS subscale scores at baseline and at the 12-week time point will be compared using paired-samples *t* tests in the aromatherapy group of this study and in the standard treatment group of our previous study. Statistical significance was set at *P*<.05. Furthermore, concerning changes in data from baseline to the 12-week time point, a two-way analysis of variance will be used analyze the interaction between the standard treatment group and the aromatherapy group. The same will be used to compare changes in SDS and POMS scores before and after smoking cessation between the historical control group and the aromatherapy group of this study.

We will also compare the successful smoking cessation rates for a period of 12 weeks from the start of treatment between the standard treatment group and the aromatherapy group. Successful smoking cessation refers to a report of smoking cessation for the past 7 days and an exhaled CO level below 7 ppm. The successful smoking cessation rate was calculated by dividing the number of subjects who successfully stopped smoking 12 weeks after smoking cessation treatment by the total number of subjects who provided consent and received the treatment, multiplied by 100 to give the percentage. Descriptive statistics on subjects’ background data will be generated. No interim analysis will be performed.

### Protection of Personal Information

All parties involved in this research shall strictly protect the personal information of the subjects in accordance with the Personal Information Protection Law. Personal information will be used to identify subjects so that accurate data can be obtained for each subject, and the personal information obtained will be managed appropriately. When information obtained from the research is published by the principal investigator or others, sufficient care will be taken to ensure that subjects are not identified.

### Early Discontinuation of the Study

The Data and Safety Monitoring Committee will recommend discontinuation of the study to the principal investigator if the experimental treatment is deemed unsafe based on safety information, including reports of serious adverse events and information outside of the study. If the committee reports to the principal investigator that the study needs to be discontinued due to safety issues or for other reasons, the principal investigator will deliberate the report and will promptly inform the secretariat in writing about the results of the deliberation. In addition to the head of the institution where the study is conducted and the IRB, the subjects will also be informed.

### Ethics Approval

This study was approved by the Kyoto Medical Center IRB on June 17, 2019 (approval No. 19-016) and has been registered in the UMIN Clinical Trials Registry (UMIN000043102).

## Results

Enrollment started on July 1, 2019. As of May 2022, 76 patients have been recruited. In the original plan, recruitment should have been finished on June 30, 2021. However, the number of subjects decreased due to the COVID-19 pandemic, and the study inclusion period was extended by 1 year (ie, until the end of June 2022) with the approval of the IRB on May 17, 2021. The observation period (ie, follow-up) will last 3 months after the enrollment of the last subject, and the study period will last for 3 years and 3 months (July 2019 to September 2022). The corresponding author will have complete access to the study data and will submit the report for publication. The results of this research will be published in international peer-reviewed journals. Both positive and negative results will be reported. In addition, the study results will be shared on the UMIN Clinical Trials Registry. Our findings will be posted on the hospital website and will be made publicly available.

## Discussion

### Principal Findings

This study is the first clinical trial study to examine the psychological effects of aromatherapy on smokers with depression. Hence, we believe that this study is of major significance. If aromatherapy can inhibit an increase in a transient propensity for depression, it can be expected to increase the rate of successful smoking cessation, which will not only reduce the risk of NCDs, but will also lead to a reduction in future medical expenses. Moreover, as aromatherapy has been reported to reduce insomnia [19], alleviate fatigue [20,21], and improve cognitive function [23], we believe that smoking cessation may have the secondary benefit of alleviating the adverse effects of smoking, such as insomnia [43,44], fatigue [45], and reduced ability to focus [44].

### Limitations

This study was designed to confirm the efficacy and safety of aromatherapy. The control group included patients in the usual care group of a previous study. This study was a pre-post single-arm study and not a double-blind randomized controlled trial. In addition, the aromatherapy survey was based on patient self-report, which may lead to inaccuracy in the frequency of inhaler use.

### Comparison With Previous Work

Smoking can cause or exacerbate negative emotions [5] and increase the risk of developing depression [6,7]. Furthermore, it has been reported that when patients with mental disorders are unstable and quit smoking, their depressive symptoms become temporarily exacerbated [8]. We have previously reported that depressive tendencies are common among smokers without a history of mental disorders [46] and that depressive tendency is the most significant factor that prevents successful smoking cessation [47]. Although aromatherapy showed potential therapeutic efficacy against depression [18], research on the efficacy of aromatherapy as an adjuvant therapy for smoking cessation is scarce. Therefore, it is significant to examine the psychological effects of aromatherapy on smokers with depression in this study.

### Conclusions

This is a pre-post single-arm study. In this study, the group treated with aromatherapy will be compared with the group that received standard treatment in a previous randomized controlled trial as the control group. If this study shows that aromatherapy has psychological benefits for patients with depression who are undergoing smoking cessation treatment, then aromatherapy could be used to improve the success rate of smoking cessation, thus reducing the risk of NCDs and lowering future medical care needs. A phase III trial is intended to be conducted to confirm the findings and correct the limitations of this study.

## Abbreviations

CO: carbon monoxide
CTCAE: Common Terminology Criteria for Adverse Events
FTND: Fagerström Test for Nicotine Dependence
IRB: Institutional Review Board
NCD: noncommunicable disease
POMS: Profile of Mood States
SDS: Zung Self-Rating Depression Scale
UMIN: University Hospital Medical Information Network

## Data Availability

Study data will be available upon reasonable request from the corresponding author.

## References

[1] World Health Organization (2013). Global Action Plan for the Prevention and Control of NCDs 2013-2020.

[2] Hartmann-Boyce J, Chepkin SC, Ye W, Bullen C, Lancaster T (2018). Nicotine replacement therapy versus control for smoking cessation. Cochrane Database Syst Rev.

[3] Cahill K, Lindson-Hawley N, Thomas KH, Fanshawe TR, Lancaster T (2016). Nicotine receptor partial agonists for smoking cessation. Cochrane Database Syst Rev.

[4] Laniado-Laborín R (2010). Smoking cessation intervention: An evidence-based approach. Postgrad Med.

[5] Hajek P, Taylor T, McRobbie H (2010). The effect of stopping smoking on perceived stress levels. Addiction.

[6] Glassman AH (1993). Cigarette smoking: Implications for psychiatric illness. Am J Psychiatry.

[7] Kendler KS, Neale MC, MacLean CJ, Heath AC, Eaves LJ, Kessler RC (1993). Smoking and major depression. A causal analysis. Arch Gen Psychiatry.

[8] Cunningham FE, Hur K, Dong D, Miller DR, Zhang R, Wei X, McCarren M, Mosholder AD, Graham DJ, Aspinall SL, Good CB (2016). A comparison of neuropsychiatric adverse events during early treatment with varenicline or a nicotine patch. Addiction.

[9] Tsoh JY, Humfleet GL, Muñoz R F, Reus VI, Hartz DT, Hall SM (2000). Development of major depression after treatment for smoking cessation. Am J Psychiatry.

[10] Howes S, Hartmann-Boyce J, Livingstone-Banks J, Hong B, Lindson N (2020). Antidepressants for smoking cessation. Cochrane Database Syst Rev.

[11] The Japanese Circulation Society, The Japan Lung Cancer Society, The Japanese Cancer Association, The Japanese Respiratory Society (2021). Standard Procedure Manual for Smoking Cessation Treatment. Version 8.1.

[12] Sharifi-Rad J, Sureda A, Tenore GC, Daglia M, Sharifi-Rad M, Valussi M, Tundis R, Sharifi-Rad M, Loizzo MR, Ademiluyi AO, Sharifi-Rad R, Ayatollahi SA, Iriti M (2017). Biological activities of essential oils: From plant chemoecology to traditional healing systems. Molecules.

[13] Lis-Balchin M (2006). Aromatherapy practice. Aromatherapy Science: A Guide for Healthcare Professionals.

[14] Ndao DH, Ladas EJ, Cheng B, Sands SA, Snyder KT, Garvin JH, Kelly KM (2012). Inhalation aromatherapy in children and adolescents undergoing stem cell infusion: Results of a placebo-controlled double-blind trial. Psychooncology.

[15] PDQ Integrative, Alternative, and Complementary Therapies Editorial Board (2002). Aromatherapy With Essential Oils (PDQ®): Health Professional Version. PDQ Cancer Information Summaries.

[16] Ali B, Al-Wabel NA, Shams S, Ahamad A, Khan SA, Anwar F (2015). Essential oils used in aromatherapy: A systemic review. Asian Pac J Trop Biomed.

[17] Burnett KM, Solterbeck LA, Strapp CM (2004). Scent and mood state following an anxiety-provoking task. Psychol Rep.

[18] Sánchez-Vidaña DI, Ngai SP, He W, Chow JK, Lau BW, Tsang HW (2017). The effectiveness of aromatherapy for depressive symptoms: A systematic review. Evid Based Complement Alternat Med.

[19] Blackburn L, Achor S, Allen B, Bauchmire N, Dunnington D, Klisovic R, Naber S, Roblee K, Samczak A, Tomlinson-Pinkham K, Chipps E (2017). The effect of aromatherapy on insomnia and other common symptoms among patients with acute leukemia. Oncol Nurs Forum.

[20] Karadag E, Samancioglu Baglama S (2019). The effect of aromatherapy on fatigue and anxiety in patients undergoing hemodialysis treatment: A randomized controlled study. Holist Nurs Pract.

[21] Kawabata N, Hata A, Aoki T (2020). Effect of aromatherapy massage on quality of sleep in the palliative care ward: A randomized controlled trial. J Pain Symptom Manage.

[22] Cheng VY, Huang C, Liao J, Hsu H, Wang S, Huang S, Guo J (2020). Combination of 3-dimensional virtual reality and hands-on aromatherapy in improving institutionalized older adults' psychological health: Quasi-experimental study. J Med Internet Res.

[23] Mehndiratta M, Pandey S, Nayak R, Alam A (2012). Posterior circulation ischemic stroke-clinical characteristics, risk factors, and subtypes in a north Indian population: A prospective study. Neurohospitalist.

[24] Rose JE, Behm FM (1994). Inhalation of vapor from black pepper extract reduces smoking withdrawal symptoms. Drug Alcohol Depend.

[25] Jang S, Lee JA, Jang B, Shin Y, Ko S, Park S (2019). Clinical effectiveness of traditional and complementary medicine interventions in combination with nicotine replacement therapy on smoking cessation: A randomized controlled pilot trial. J Altern Complement Med.

[26] Sayette MA, Marchetti MA, Herz RS, Martin LM, Bowdring MA (2019). Pleasant olfactory cues can reduce cigarette craving. J Abnorm Psychol.

[27] López V, Nielsen B, Solas M, Ramírez MJ, Jäger AK (2017). Exploring pharmacological mechanisms of lavender (Lavandula angustifolia) essential oil on central nervous system targets. Front Pharmacol.

[28] Zhou W, Fukumoto S, Yokogoshi H (2009). Components of lemon essential oil attenuate dementia induced by scopolamine. Nutr Neurosci.

[29] Umezu T, Sakata A, Ito H (2001). Ambulation-promoting effect of peppermint oil and identification of its active constituents. Pharmacol Biochem Behav.

[30] Agatonovic-Kustrin S, Kustrin E, Gegechkori V, Morton D (2020). Anxiolytic terpenoids and aromatherapy for anxiety and depression. Adv Exp Med Biol.

[31] Dyer J, Cleary L, Ragsdale-Lowe M, McNeill S, Osland C (2014). The use of aromasticks at a cancer centre: A retrospective audit. Complement Ther Clin Pract.

[32] Stringer J, Donald G (2011). Aromasticks in cancer care: An innovation not to be sniffed at. Complement Ther Clin Pract.

[33] Fukuda K, Kobayashi S (2011). A Manual of the Japanese Version of the Zung SDS (Self-Rating Depression Scale)-The Augmented Edition [document in Japanese].

[34] National Cancer Institute (2010). Common Terminology Criteria for Adverse Events (CTCAE) Version 4.03.

[35] Zung WW (1965). A self-rating depression scale. Arch Gen Psychiatry.

[36] Dani JA, Heinemann S (1996). Molecular and cellular aspects of nicotine abuse. Neuron.

[37] Sun J (2007). D-Limonene: Safety and clinical applications. Altern Med Rev.

[38] Cheallaigh AN, Mansell DJ, Toogood HS, Tait S, Lygidakis A, Scrutton NS, Gardiner JM (2018). Chemoenzymatic synthesis of the intermediates in the peppermint monoterpenoid biosynthetic pathway. J Nat Prod.

[39] Gostner JM, Ganzera M, Becker K, Geisler S, Schroecksnadel S, Überall F, Schennach H, Fuchs D (2014). Lavender oil suppresses indoleamine 2,3-dioxygenase activity in human PBMC. BMC Complement Altern Med.

[40] Dosoky NS, Satyal P, Barata LM, da Silva JKR, Setzer WN (2019). Volatiles of black pepper fruits (Piper nigrum L). Molecules.

[41] McNair DM (1992). Profile of Mood States.

[42] Brown RA, Lejuez CW, Kahler CW, Strong DR, Zvolensky MJ (2005). Distress tolerance and early smoking lapse. Clin Psychol Rev.

[43] Clinical Practice Guideline Treating Tobacco Use and Dependence 2008 Update Panel‚ Liaisons‚ and Staff (2008). A clinical practice guideline for treating tobacco use and dependence: 2008 update. A US Public Health Service report. Am J Prev Med.

[44] Barua RS, Rigotti NA, Benowitz NL, Cummings KM, Jazayeri M, Morris PB, Ratchford EV, Sarna L, Stecker EC, Wiggins BS (2018). 2018 ACC Expert Consensus Decision Pathway on Tobacco Cessation Treatment: A report of the American College of Cardiology Task Force on Clinical Expert Consensus Documents. J Am Coll Cardiol.

[45] Anderson JE, Jorenby DE, Scott WJ, Fiore MC (2002). Treating tobacco use and dependence: An evidence-based clinical practice guideline for tobacco cessation. Chest.

[46] Hasegawa K, Terashima S, Satoh N, Inoue M, Wada H, Itoh C, Iida Y, Yamakage H, Shimatsu A, Takahashi Y (2008). [Depressive state of patients on their initial visit to a smoking cessation clinic] [Article in Japanese]. Jpn J Smok Control Sci.

[47] Wada H, Hasegawa K, Terashima S, Satoh N, Inoue M, Iida Y, Yamakage H, Kitaoka S, Morimoto T, Fujita M, Shimatsu A, Takahashi Y (2008). [Self-rating depression scale score is a strong independent predictor of smoking cessation outcomes] [Article in Japanese]. Jpn J Smok Control Sci.

